# Causal relationship between rheumatoid arthritis and bronchiectasis: a bidirectional mendelian randomization study

**DOI:** 10.1186/s13075-024-03336-3

**Published:** 2024-05-23

**Authors:** Zehu Chen, Xuegang Li, Honglei Shi, Yiying Huang, Jing Liu

**Affiliations:** 1 Department of Respiratory and Critical Care Medicine, the Fifth Affiliated Hospital of Sun Yat-sen University, Zhuhai, China; 2 Department of Rheumatology, the Fifth Affiliated Hospital of Sun Yat-sen University, Zhuhai, China; 3 Department of Respiratory and Critical Care Medicine, Affiliated Beijing Chaoyang Hospital of Capital Medical University, Beijing, China

**Keywords:** Rheumatoid arthritis, Bronchiectasis, Mendelian randomization, Genome-wide association study, Single-nucleotide polymorphisms

## Abstract

**Background:**

Epidemiological observational studies have elucidated a correlation between rheumatoid arthritis (RA) and bronchiectasis. However, the causal nature of this association remains ambiguous. To clarify this potential causal linkage, we conducted a two-sample Mendelian randomization (MR) analysis to explore the bidirectional causality between RA and bronchiectasis.

**Methods:**

Summary statistics for RA and bronchiectasis were obtained from the IEU OpenGWAS database We employed various methods, including inverse variance weighting (IVW), MR-Egger, weighted median, weighted mode, and simple mode, to explore potential causal links between RA and bronchiectasis. Additionally, a series of sensitivity studies, such as Cochran’s Q test, MR Egger intercept test, and leave-one-out analysis, were conducted to assess the MR analysis’s accuracy further.

**Results:**

In the forward MR analysis, the primary analysis indicated that a genetic predisposition to RA correlated with an increased risk of bronchiectasis in European populations (IVW odds ratio (OR): 1.28, 95% confidence interval (CI): 1.20–1.37, *p* = 1.18E-13). Comparable results were noted in the East Asian subjects (IVW OR: 1.55, 95% CI: 1.30–1.34, *p* = 8.33E-07). The OR estimates from the other four methods were consistent with those obtained from the IVW method. Sensitivity analysis detected no evidence of horizontal pleiotropy or heterogeneity. Conversely, in the reverse MR analysis, we found no evidence to support a genetic causality between bronchiectasis and RA in either European or East Asian populations.

**Conclusion:**

This study indicates that genetic predisposition to RA correlates with a heightened risk of bronchiectasis in both European and East Asian populations. These results imply that routine screening for bronchiectasis in RA patients could be beneficial, and effective management of RA may contribute to a reduced risk of bronchiectasis. Future research should aim to clarify the underlying mechanisms linking these two conditions.

**Supplementary Information:**

The online version contains supplementary material available at 10.1186/s13075-024-03336-3.

## Background

Rheumatoid arthritis (RA) is a chronic systemic autoimmune disorder characterized by progressive joint damage and extra-articular manifestations, potentially leading to permanent disability and heightened mortality [1]. A meta-analysis of 67 rheumatoid arthritis cohort studies across 41 countries indicated a pooled prevalence of 0.46% [2]. The most common extra-articular manifestation of RA is lung involvement, which can affect up to 60% of patients with RA during the disease course [3]. Bronchiectasis is an established extra-articular manifestation of RA, which presents as irreversible damage to the bronchi along with widening and thickening resulting in exuberant mucus production [4]. Approximately 30% of RA patients demonstrate bronchiectasis on High-Resolution Computed Tomography, which can be asymptomatic and may precede or follow the development of RA [5–7]. RA-associated bronchiectasis significantly reduces quality of life and increases the risk of mortality [4, 8]. Consequently, it is imperative to investigate the relationship and pathogenesis between RA and bronchiectasis.

The link between bronchiectasis and RA has been a subject of extensive research over several decades. Numerous studies indicate that RA may contribute to the risk of developing bronchiectasis. For instance, a systematic review and meta-analysis revealed an elevated prevalence of both radiological and symptomatic bronchiectasis among RA patients relative to the general population [9]. Conversely, evidence also suggests that bronchiectasis could be a risk factor for RA. A prospective study observed that 50% of bronchiectasis patients with positive rheumatoid factor and high anti-cyclic citrullinated peptide levels developed RA within a 12-month period [10].

These observational studies could be influenced by potential confounders and reverse causation, which makes it difficult to ascertain the causality between bronchiectasis and RA. Mendelian randomization (MR), a statistical approach employing genetic variation as an instrumental variable (IV), evaluates whether the observational association between exposure factors and outcomes aligns with a causal effect [11]. It avoided confounding and reverse causality because genetic variants were identified at the time of conception [12]. Consequently, our study took advantage of MR and investigated the bidirectional causal relationship between bronchiectasis and RA.

## Materials and methods

### Study design

**Fig. 1:**
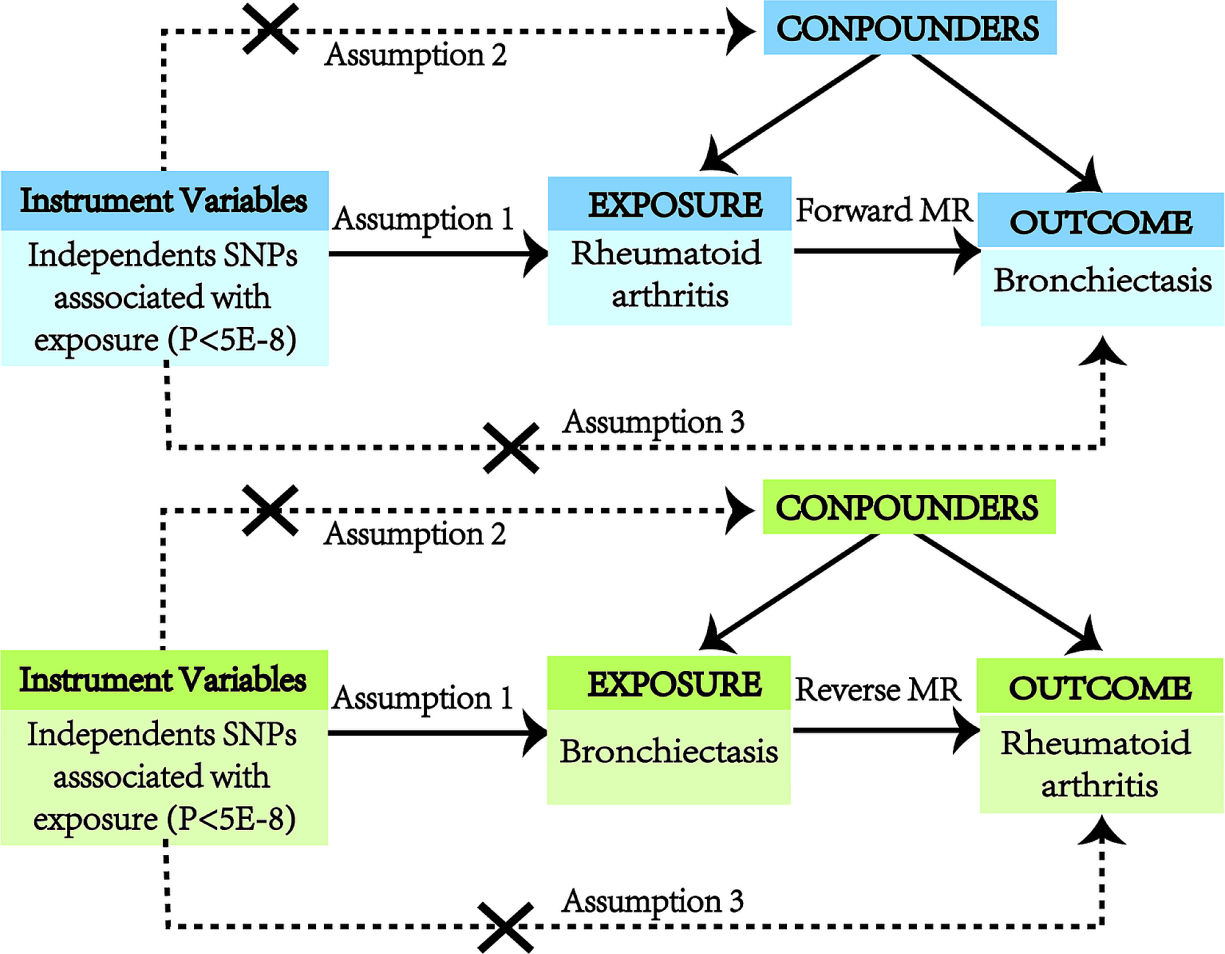
Study design for our Mendelian randomization

An overview of the bidirectional MR study design is shown in Fig. 1. First, a two-sample MR analysis was conducted, treating RA as the exposure and bronchiectasis as the outcome. Subsequently, the analysis was inverted to investigate bronchiectasis as the exposure and RA as the outcome. The validity of MR analysis hinges on three critical assumptions [13]: (1) There is a strong association between the instrumental variables and the exposure; (2) Each IV is not associated with any confounding variables; (3) Each IV is associated with the outcome solely through the exposure, and there are no alternative pathways for the association.

### GWAS data sources

The datasets for this MR study were obtained from the publicly accessible IEU Open GWAS database (https://gwas.mrcieu.ac.uk/, accessed on 19 November 2023). Summary statistics for RA were obtained from a GWAS including 417,256 European subjects (8,255 cases and 409,001 controls) and another encompassing 178,616 individuals (2,888 cases and 440,263 controls) from East Asian populations. Similarly, bronchiectasis data were derived from a GWAS including 443,151 European subjects (5,348 cases and 173,268 controls) and an additional study involving 162,044 East Asian individuals (241 cases and 161,803 controls). Comprehensive details of all GWAS used in our study are detailed in Table 1.

**Table 1:** Details of GWAS included in Mendelian randomization analysis

Traits	GWAS ID	Year	Ancestor	Sample Size	Number of SNP
RA	ebi-a-GCST90018910	2021	European	417,256	24,175,266
Bronchiectasis	ebi-a-GCST90018801	2021	European	443,151	24,189,609
RA	ebi-a-GCST90018690	2021	East Asian	178,616	12,454,695
Bronchiectasis	ebi-a-GCST90018581	2021	East Asian	162,044	12,451,975

RA, rheumatoid arthritis; GWAS, Genome Wide Association Study; SNP, single nucleotide polymorphism;

### Instrumental variable selection

To ensure the validity of our genetic IVs and satisfy the three core MR assumptions, we employed a comprehensive set of quality control techniques. Firstly, single nucleotide polymorphisms (SNPs) associated with the exposure were identified using a genome-wide significance threshold of *p* < 5E–08. In cases where the number of SNPs meeting this criterion is inadequate, SNPs with a relaxed significance threshold (*p* < 5E–06) may be considered as IVs. Secondly, to reduce linkage disequilibrium, the identified exposure SNPs were clumped (r² = 0.001, window = 10 Mb, *p* -value threshold = 5E-8) utilizing the 1000 Genomes European dataset as a reference. Thirdly, we also excluded SNPs with palindromic intermediate allele frequencies and incompatible alleles [14]. Fourthly, each SNP’s secondary phenotype was assessed using the PhenoScanner database (http://www.phenoscanner.medschl.cam.ac.uk/) with a *p* -value threshold of *p* < 1E–05, and SNPs linked to confounders were excluded to mitigate pleiotropic effects. Finally, we ensured that the F statistic of all IVs was greater than the threshold 10 to minimize weak instrument bias. The F statistic was calculated using the formula F = R2(N-K-1)/K(1-R2) [15].

### Statistical analysis

This study employed multiple complementary methods to explore the causal relationship between bronchiectasis and RA. These methods included the inverse variance weighted method, the weighted median approach, the MR-Egger regression, the simple mode and weighted mode. The IVW method, used as the primary analysis for causal estimates, was most precise when all instrumental variables are valid [16]. The weighted median approach offered a consistent estimate of the causal effect when at least half of the SNPs serve as effective IVs [17]. The MR-Egger regression was used to confirm whether horizontal pleiotropy of IVs exists, and its intercept represents the effect estimate of horizontal pleiotropy [18]. The simple mode and weighted mode were performed as complementary analyses.

Additionally, we performed sensitivity analyses to assess the dependability of our findings. The MR-Egger intercept was used to determine directional horizontal pleiotropy [19]. Subsequently, the Mendelian randomization pleiotropy residual sum and outlier (MR-PRESSO) test was applied to detect potential horizontal pleiotropy and correct it by removing outliers [20]. The Cochrane Q test was used to evaluate heterogeneity between SNPs [21]. Furthermore, the leave-one-out analysis was used to investigate whether the genetic causal relationship between exposures and outcomes was influenced by a single SNP [22]. All statistical analyses were performed using the “TwoSampleMR” and “MR-PRESSO” packages in R software (version 4.3.1).

## Results

### Forward MR analyses of the effects of RA on bronchiectasis

Following a rigorous selection process for eligible IVs and the exclusion of potential pleiotropic SNPs, 24 SNPs for RA in European populations and 31 SNPs for RA in East Asian populations were finally identified as IVs in the MR analysis. The F-statistics of the screened SNPs were all > 10 to avoid bias caused by weak IVs. The information of these genetic variants utilized in the MR analyses is detailed in Supplementary Tables 1–2.

**Fig. 2:**
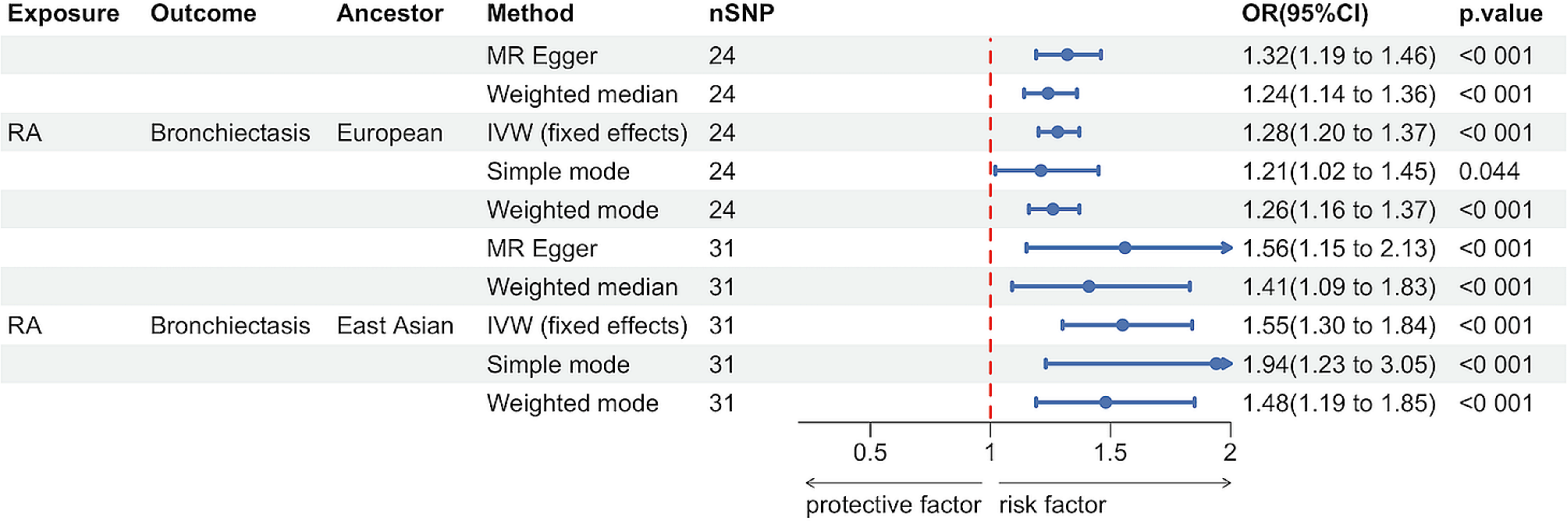
Mendelian randomization for the association of rheumatoid arthritis on bronchiectasis

Figure 2 illustrates that the MR results support a causal link between genetic susceptibility to RA and an increased risk of bronchiectasis. Among individuals of European ancestry, the primary analysis revealed a significant causal association (IVW odds ratio (OR): 1.28, 95% confidence interval (CI): 1.20–1.37, *p* = 1.18E-13). Similar findings were observed in the East Asian population (IVW OR: 1.55, 95% CI: 1.30–1.34, *p* = 8.33E-07). This result was broadly consistent with results obtained using other MR methods including the weighted median approach, the MR-Egger regression, the simple mode and weighted mode.

In the sensitivity analysis, the Cochran Q test revealed no heterogeneity (*p* -value > 0.05), and the MR-Egger regression indicated no evidence of pleiotropy (intercept *p* -value > 0.05), as detailed in Table 2. Additionally, the leave-one-out analysis suggested that the causal effect of RA on bronchiectasis was not driven by any single SNP (Supplementary Figs. 1–2).

**Table 2:** Sensitivity analysis of the associations between rheumatoid arthritis and bronchiectasis

Exposures	Outcomes	Ancestor	Heterogeneity test (MR-Egger)		Heterogeneity test (IVW)		Pleiotropy test (MR-Egger)	
			Cochran’s Q	*P*	Cochran’s Q	*P*	Intercept	*P*
RA	Bronchiectasis	European	20.20	0.57	20.72	0.59	-0.007	0.48
RA	Bronchiectasis	East Asian	31.77	0.33	31.78	0.38	-0.003	0.95
Bronchiectasis	RA	European	1.51	0.91	3.76	0.71	-0.0318	0.19
Bronchiectasis	RA	East Asian	1.38	0.71	4.39	0.36	0.0269	0.18

RA, rheumatoid arthritis; IVW, inverse variance weighted

### Reverse MR analyses of the effects of bronchiectasis on RA

After using strict criteria to rule out SNPs, 7 SNPs for bronchiectasis in European populations and 5 SNPs for bronchiectasis in Asian populations were identified as IVs in the MR analysis. The F-statistics of the screened SNPs were all > 10 to avoid bias caused by weak IVs. The information of these genetic variants utilized in the MR analyses is detailed in Supplementary Tables 3–4.

**Fig. 3:**
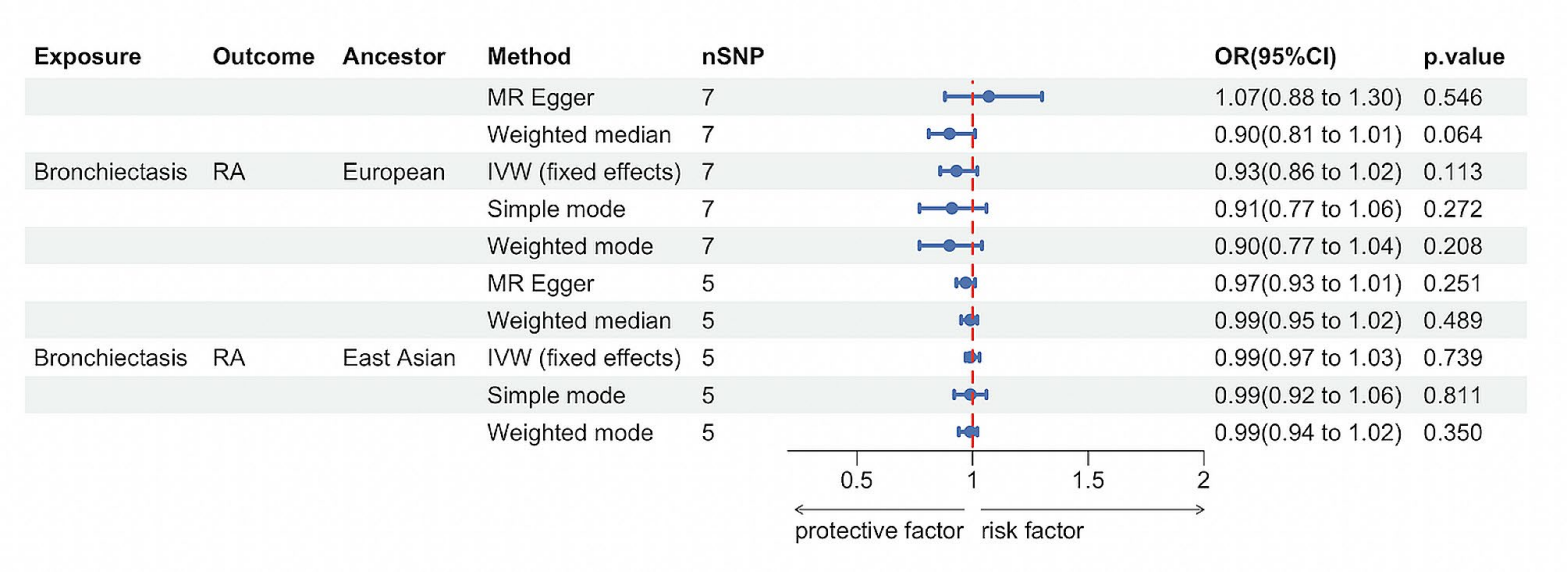
Mendelian randomization for the association of bronchiectasis on rheumatoid arthritis

As shown in Fig. 3, there were no causal effects of bronchiectasis on RA either in European ancestry (IVW OR: 0.93, 95% CI: 0.86–1.02, *p* = 0.113) or East Asian populations (IVW OR: 0.99, 95% CI: 0.97–1.03, *p* = 0.74). This absence of causal effects was consistent across various MR methods including the weighted median approach, the MR-Egger regression, the simple mode and weighted mode. The sensitivity analysis did not find heterogeneity (*p* -value of Cochran Q > 0.05) and pleiotropy (*p* -value for intercept > 0.05) (Table 2). Leave-one-out analysis showed that no outlier SNP was detected. (Supplementary Figs. 3–4).

## Discussion

For the first time, we conducted a bidirectional two-sample MR analysis to investigate the causal effect between RA and bronchiectasis. The results revealed a causal association between genetic susceptibility to RA and an increased risk of bronchiectasis. Conversely, no evidence was found to suggest a causal relationship between bronchiectasis and an increased risk of RA. The findings of this study offer significant insights into the potential causal connection between these two conditions.

Epidemiological research has consistently shown a link between rheumatoid arthritis and bronchiectasis. For instance, a cross-sectional study indicated that RA might contribute to more severe and extensive bronchiectasis [23]. Additionally, evidence from a retrospective cohort study suggested that RA disease duration is associated with higher risk of having respiratory symptoms (not specific to bronchiectasis) [24]. These results are consistent with our forward MR analysis. However, our reverse MR analysis provided contrasting evidence, diverging from observational studies. A recent meta-analysis revealed that bronchiectasis preceded RA in 53 of 69 cases, indicating that bronchiectasis may contribute to the development of RA [9]. Furthermore, a prospective study observed that 50% of bronchiectasis patients with positive rheumatoid factor and high anti-cyclic citrullinated peptide levels developed RA within a 12-month period [10]. A potential explanation for the differences between the results of observational studies and our reverse MR analysis might be that the previously observed results were either coincidental or influenced by unidentified confounding factors.

Several hypothesis have been put forward to explain the mechanisms underlying the development of bronchiectasis in RA [25]. Primarily, the onset of bronchiectasis in RA may be linked to RA-related autoantibodies. Aubart et al. demonstrated that elevated levels of anti-cyclic citrullinated peptide antibodies (ACPA) correlated with the occurrence of pulmonary diseases like bronchiectasis in RA patients [26]. Additionally, Attar et al. reported that patients with RA who were positive for ACPA exhibited an increased risk of bronchiectasis [27]. Collectively, these studies implied that autoantibodies substantially may contribute to bronchiectasis in RA patients. Secondly, the likelihood that recurrent infections related to chronic immune suppression may predispose patients with rheumatoid arthritis to bronchiectasis [8]. For instance, patients with RA and bronchiectasis have frequent lower respiratory tract infections, and these infections were associated with the utilization of biologic disease-modifying antirheumatic drugs (DMARD) [28]. Consequently, it is hypothesized that the recurrent infections linked to DMARD use in RA patients might contribute to an increased risk of concurrent bronchiectasis. Lastly, genetic factors may also play a role in the association between RA and bronchiectasis. A family-based association study highlighted that cystic fibrosis transmembrane conductance regulator (CFTR) gene mutations in RA patients are indicators of associated bronchiectasis risk [29]. However, research indicates that only 15.4% of patients diagnosed with both bronchiectasis and rheumatoid arthritis display mutations in the CFTR gene [30], suggesting that these mutations may have a minimal impact.

There are several advantages to this study. Firstly, we used a MR analysis that is not affected by confounders and reverse causation compared with observational studies. Secondly, the bidirectional approach facilitates the exploration of causal relationships in both directions between RA and bronchiectasis. Additionally, the validation of our findings across European and East Asian datasets enhances the credibility of our conclusions. However, there are still limitations. Firstly, the MR results are primarily based on European and East Asian populations, and the applicability of these findings to other populations requires further investigation. Secondly, the absence of demographic data such as gender and age in the public databases precluded the possibility of conducting additional subgroup analyses. Furthermore, the SNPs used in the analysis may be associated with other traits due to genetic polymorphisms, potentially impacting the accuracy of causal inference.

## Conclusions

In summary, this study utilized a bidirectional two-sample MR approach to assess the causal relationship between RA and bronchiectasis. It established a causal link between genetic predisposition to RA and an elevated risk of bronchiectasis, while not finding a similar association for genetic predisposition to bronchiectasis increasing the risk of RA. These findings suggest that routine screening for bronchiectasis in RA patients, coupled with effective RA management, could help mitigate the risk of developing bronchiectasis. Additionally, it is crucial to carry out a placebo-controlled randomized trial in patients with RA associated bronchiectasis to identify treatment strategies that improve prognosis for this subgroup. Furthermore, future research should focus on clarifying the underlying mechanisms that link RA and bronchiectasis.

### Electronic supplementary material

Below is the link to the electronic supplementary material.

Supplementary Material 1

## Data Availability

Publicly available datasets can be found here: the Integrative Epidemiology Unit (IEU) GWAS database (https://gwas.mrcieu.ac.uk).

## Abbreviations

RA: Rheumatoid arthritis
MR: Mendelian randomization
SNP: Single-nucleotide polymorphism
GWAS: Genome-wide association
IVW: Inverse-variance-weighted
OR: Odds ratio
CI: confidence interval
MRPRESSO: Mendelian randomization pleiotropy residual sum and outlier
ACPA: anti-cyclic citrullinated peptide antibodies
CFTR: cystic fibrosis transmembrane conductance regulator study
