# Supplementary material for: Causal relationship between rheumatoid arthritis and bronchiectasis: a bidirectional mendelian randomization study
Source: Arthritis Res Ther. 2024 May 23;26:104. doi: 10.1186/s13075-024-03336-3 (PMC11112812; doi:10.1186/s13075-024-03336-3)
Supplement: Supplementary file 1 — Supplementary Material 1 [file 13075_2024_3336_MOESM1_ESM.docx]

# The supplementary materials include:

# 1) Supplementary Tables 1-4

# 2) Supplementary Figures 1-4

# Supplementary Tables 1-4

**Supplementary Table 1.** Characteristics of the RA-associated genetic instrumental variables included in the MR study (European populations).

| **SNP** | **Chr** | **Pos** | **EA** | **OA** | **EAF** | **Se** | ***P*** | **R²** | **F** |
| --- | --- | --- | --- | --- | --- | --- | --- | --- | --- |
| rs10261758 | 7 | 50353192 | A | G | 0.710671 | 0.0143 | 1.47E-08 | 7.69E-05 | 32.0845447 |
| rs10797431 | 1 | 2501222 | T | G | 0.403594 | 0.0129 | 1.06E-08 | 7.89E-05 | 32.9065186 |
| rs11889341 | 2 | 191943742 | T | C | 0.249601 | 0.0146 | 2.71E-17 | 0.00017062 | 71.2055135 |
| rs142144003 | 6 | 32433702 | A | G | 0.111153 | 0.0207 | 1.09E-88 | 0.00095495 | 398.839508 |
| rs1611318 | 6 | 29902240 | C | A | 0.76162 | 0.0185 | 1.07E-11 | 0.00011098 | 46.3133207 |
| rs1776616 | 10 | 31350494 | G | A | 0.664992 | 0.0139 | 2.41E-09 | 8.59E-05 | 35.8273734 |
| rs2156698 | 11 | 128488322 | A | G | 0.4561 | 0.0131 | 1.44E-08 | 7.75E-05 | 32.3420745 |
| rs2618444 | 8 | 11338370 | C | A | 0.387409 | 0.0146 | 6.71E-12 | 0.00011355 | 47.3831938 |
| rs28362859 | 6 | 44228815 | C | A | 0.100049 | 0.0205 | 9.90E-13 | 0.00012238 | 51.0699515 |
| rs34536443 | 19 | 10463118 | C | G | 0.0427749 | 0.0427 | 3.03E-08 | 7.36E-05 | 30.7283106 |
| rs35139284 | 6 | 32561370 | T | C | 0.319092 | 0.0149 | 1.00E-200 | 0.00312292 | 1307.13166 |
| rs35511257 | 6 | 32545392 | C | G | 0.074625 | 0.0408 | 4.38E-64 | 0.0006828 | 285.095237 |
| rs362531 | 6 | 29530455 | A | G | 0.0614836 | 0.0252 | 3.47E-17 | 0.00016975 | 70.8399382 |
| rs3757387 | 7 | 128576086 | C | T | 0.34443 | 0.0147 | 1.47E-13 | 0.00013079 | 54.5786638 |
| rs56376587 | 18 | 77160235 | C | A | 0.429311 | 0.0133 | 3.77E-09 | 8.35E-05 | 34.8364546 |
| rs5754100 | 22 | 21916166 | C | T | 0.244139 | 0.015 | 9.20E-09 | 7.88E-05 | 32.8709536 |
| rs5757628 | 22 | 39749981 | A | G | 0.427512 | 0.0157 | 7.58E-10 | 9.07E-05 | 37.8575815 |
| rs62395272 | 6 | 31394424 | T | C | 0.110971 | 0.0206 | 6.60E-53 | 0.00055865 | 233.228121 |
| rs6679677 | 1 | 114303808 | A | C | 0.112873 | 0.0254 | 2.54E-33 | 0.00034567 | 144.282912 |
| rs71508903 | 10 | 63779871 | T | C | 0.210869 | 0.0157 | 2.54E-08 | 7.39E-05 | 30.8483247 |
| rs7731626 | 5 | 55444683 | A | G | 0.278947 | 0.0157 | 9.77E-18 | 0.00017534 | 73.1729626 |
| rs9277814 | 6 | 33101675 | G | A | 0.510227 | 0.0145 | 6.12E-20 | 0.00019918 | 83.1236919 |
| rs9469053 | 6 | 31755776 | G | A | 0.0454311 | 0.0312 | 1.22E-20 | 0.00020801 | 86.8118065 |
| rs9494894 | 6 | 138228520 | C | T | 0.0417083 | 0.031 | 2.48E-08 | 7.43E-05 | 30.9996017 |

**Supplementary Table 2**. Characteristics of the RA-associated genetic instrumental variables included in the MR study (East Asian populations)

| **SNP** | **Chr** | **Pos** | **EA** | **OA** | **EAF** | **Se** | ***P*** | **R²** | **F** |
| --- | --- | --- | --- | --- | --- | --- | --- | --- | --- |
| rs10821944 | 10 | 63785089 | T | G | 0.633689 | 0.0209848 | 5.63E-09 | 0.00019008 | 33.9571978 |
| rs111335405 | 6 | 32655722 | G | A | 0.179899 | 0.0282376 | 1.88E-118 | 0.00298857 | 535.401055 |
| rs114665205 | 6 | 31312423 | G | A | 0.00893145 | 0.118571 | 1.83E-10 | 0.00022747 | 40.6394038 |
| rs11889341 | 2 | 191943742 | T | C | 0.30631 | 0.0219097 | 6.64E-09 | 0.00018829 | 33.6373679 |
| rs12439845 | 15 | 41211827 | C | T | 0.698101 | 0.0220292 | 5.83E-07 | 0.00013977 | 24.9675009 |
| rs12805524 | 11 | 128395947 | G | A | 0.187273 | 0.026557 | 3.20E-06 | 0.00012144 | 21.6937373 |
| rs1362076 | 6 | 29441169 | T | G | 0.077025 | 0.0386606 | 1.33E-17 | 0.00040826 | 72.9508755 |
| rs199949106 | 6 | 30692754 | T | C | 0.170129 | 0.0272177 | 3.31E-06 | 0.00012108 | 21.6300131 |
| rs2069235 | 22 | 39747780 | A | G | 0.858095 | 0.0290813 | 1.25E-06 | 0.00013156 | 23.500793 |
| rs2235369 | 6 | 14118505 | A | G | 0.149922 | 0.028283 | 8.46E-07 | 0.00013575 | 24.2502081 |
| rs2618476 | 8 | 11352541 | C | T | 0.696507 | 0.0227152 | 6.54E-08 | 0.00016343 | 29.1953049 |
| rs2647192 | 1 | 17407207 | A | C | 0.395463 | 0.0208692 | 1.13E-07 | 0.00015749 | 28.1340139 |
| rs28362859 | 6 | 44228815 | C | A | 0.219096 | 0.024848 | 4.31E-12 | 0.00026855 | 47.9799505 |
| rs2841280 | 14 | 105393556 | C | G | 0.628702 | 0.0210249 | 1.19E-06 | 0.00013203 | 23.585131 |
| rs34493748 | 6 | 33001771 | C | T | 0.0467224 | 0.0493405 | 5.61E-15 | 0.00034159 | 61.0339483 |
| rs3910172 | 10 | 64067508 | T | C | 0.894138 | 0.0334478 | 1.55E-07 | 0.00015408 | 27.5245497 |
| rs4350841 | 21 | 45723628 | C | T | 0.384673 | 0.0207707 | 1.24E-07 | 0.00015651 | 27.9588318 |
| rs4728142 | 7 | 128573967 | A | G | 0.104298 | 0.0331959 | 6.89E-10 | 0.00021299 | 38.0518582 |
| rs564419538 | 6 | 32008417 | C | G | 0.248348 | 0.0267614 | 1.00E-25 | 0.00061521 | 109.952582 |
| rs58107865 | 4 | 109061618 | C | G | 0.25341 | 0.0261524 | 4.21E-08 | 0.00016821 | 30.0488615 |
| rs6461145 | 7 | 15026872 | A | G | 0.561841 | 0.0203244 | 7.35E-07 | 0.00013727 | 24.5208459 |
| rs6570194 | 6 | 138242638 | C | A | 0.0723521 | 0.0395092 | 1.51E-07 | 0.00015438 | 27.5780594 |
| rs67140765 | 6 | 31380787 | T | G | 0.129854 | 0.032247 | 1.12E-33 | 0.00081839 | 146.29604 |
| rs7172228 | 15 | 53059683 | T | G | 0.049464 | 0.0474625 | 2.26E-06 | 0.00012517 | 22.3595715 |
| rs741242 | 12 | 4249427 | C | A | 0.0597256 | 0.0438163 | 3.62E-06 | 0.00012012 | 21.4581223 |
| rs76153210 | 6 | 44284508 | T | C | 0.234602 | 0.0247696 | 2.95E-08 | 0.00017209 | 30.742458 |
| rs76895136 | 5 | 79577762 | G | A | 0.079421 | 0.0386003 | 2.88E-06 | 0.00012257 | 21.8962376 |
| rs78897192 | 1 | 161595080 | T | A | 0.664078 | 0.0253716 | 4.51E-07 | 0.00014254 | 25.4625892 |
| rs7990 | 6 | 32609965 | A | C | 0.312868 | 0.0229544 | 1.39E-142 | 0.00360564 | 646.348913 |
| rs9258276 | 6 | 29731791 | T | C | 0.180535 | 0.0263485 | 2.89E-15 | 0.00034889 | 62.3390722 |
| rs9269271 | 6 | 32539107 | C | T | 0.239225 | 0.0289542 | 1.45E-36 | 0.00089219 | 159.499807 |

**Supplementary Table 3.** Characteristics of the bronchiectasis-associated genetic instrumental variables included in the MR study (European populations)

| **SNP** | **Chr** | **Pos** | **EA** | **OA** | **EAF** | **Se** | ***P*** | **R²** | **F** |
| --- | --- | --- | --- | --- | --- | --- | --- | --- | --- |
| rs111833721 | 4 | 161347062 | A | G | 0.00786788 | 0.1562 | 1.76E-06 | 5.16E-05 | 22.8522141 |
| rs115965237 | 4 | 66709250 | A | G | 0.0575501 | 0.0568 | 3.85E-06 | 4.81E-05 | 21.3091803 |
| rs117371606 | 8 | 3836249 | G | A | 0.0115674 | 0.1301 | 1.10E-06 | 5.36E-05 | 23.7402315 |
| rs148974339 | 14 | 28994354 | A | C | 0.00960383 | 0.1401 | 2.87E-06 | 4.94E-05 | 21.9111208 |
| rs62124557 | 2 | 18290556 | T | C | 0.242085 | 0.0311 | 9.17E-07 | 5.42E-05 | 24.0130429 |
| rs6696687 | 1 | 168677830 | G | A | 0.0800083 | 0.044 | 2.98E-06 | 4.92E-05 | 21.8130472 |
| rs9892348 | 17 | 9357383 | C | T | 0.294374 | 0.0355 | 1.34E-06 | 5.28E-05 | 23.3927847 |

**Supplementary Table 4.** Characteristics of the bronchiectasis-associated genetic instrumental variables included in the MR study (East Asian populations)

| **SNP** | **Chr** | **Pos** | **EA** | **OA** | **EAF** | **Se** | ***P*** | **R²** | **F** |
| --- | --- | --- | --- | --- | --- | --- | --- | --- | --- |
| rs2315577 | 17 | 9704297 | G | A | 0.526117 | 0.0927342 | 4.05E-06 | 0.00013107 | 21.2415326 |
| rs3793860 | 10 | 61944987 | A | G | 0.237888 | 0.110386 | 2.18E-06 | 0.0001384 | 22.4292393 |
| rs4086666 | 11 | 13875937 | G | T | 0.00047928 | 4.66929 | 6.79E-07 | 0.00015224 | 24.6729898 |
| rs6084813 | 20 | 4617875 | T | C | 0.318951 | 0.114407 | 4.06E-06 | 0.00013103 | 21.235218 |
| rs999135 | 19 | 57696385 | A | G | 0.253844 | 0.10694 | 2.53E-06 | 0.00013664 | 22.1450502 |

# Supplementary Figures 1-4


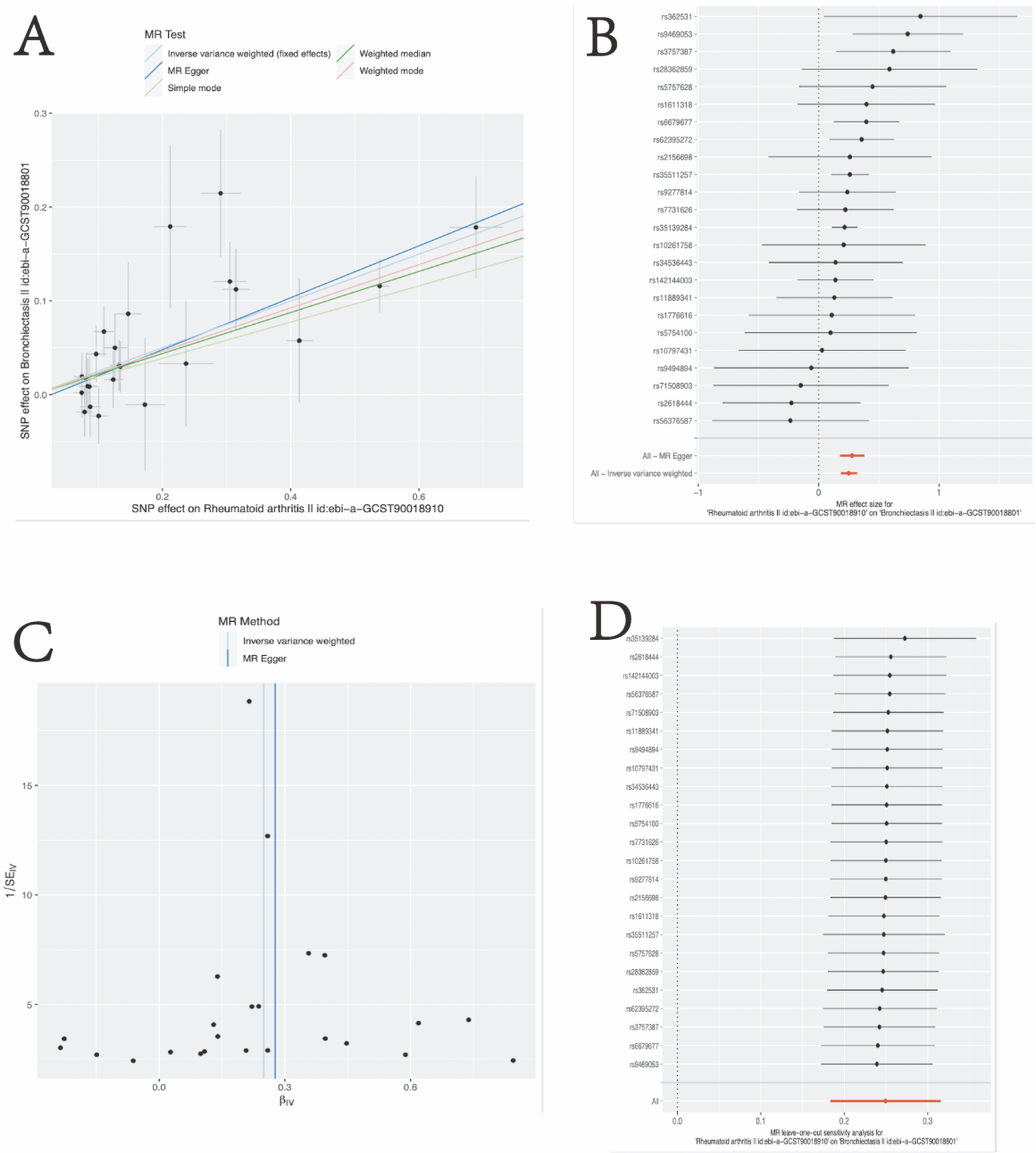


**Supplementary Figure 1. MR sensitivity analysis of RA on bronchiectasis in European populations**

Scatter plot (A), forest plot (B), funnel plot (C) and leave-one-out sensitivity analysis (D) of the effect of RA on bronchiectasis


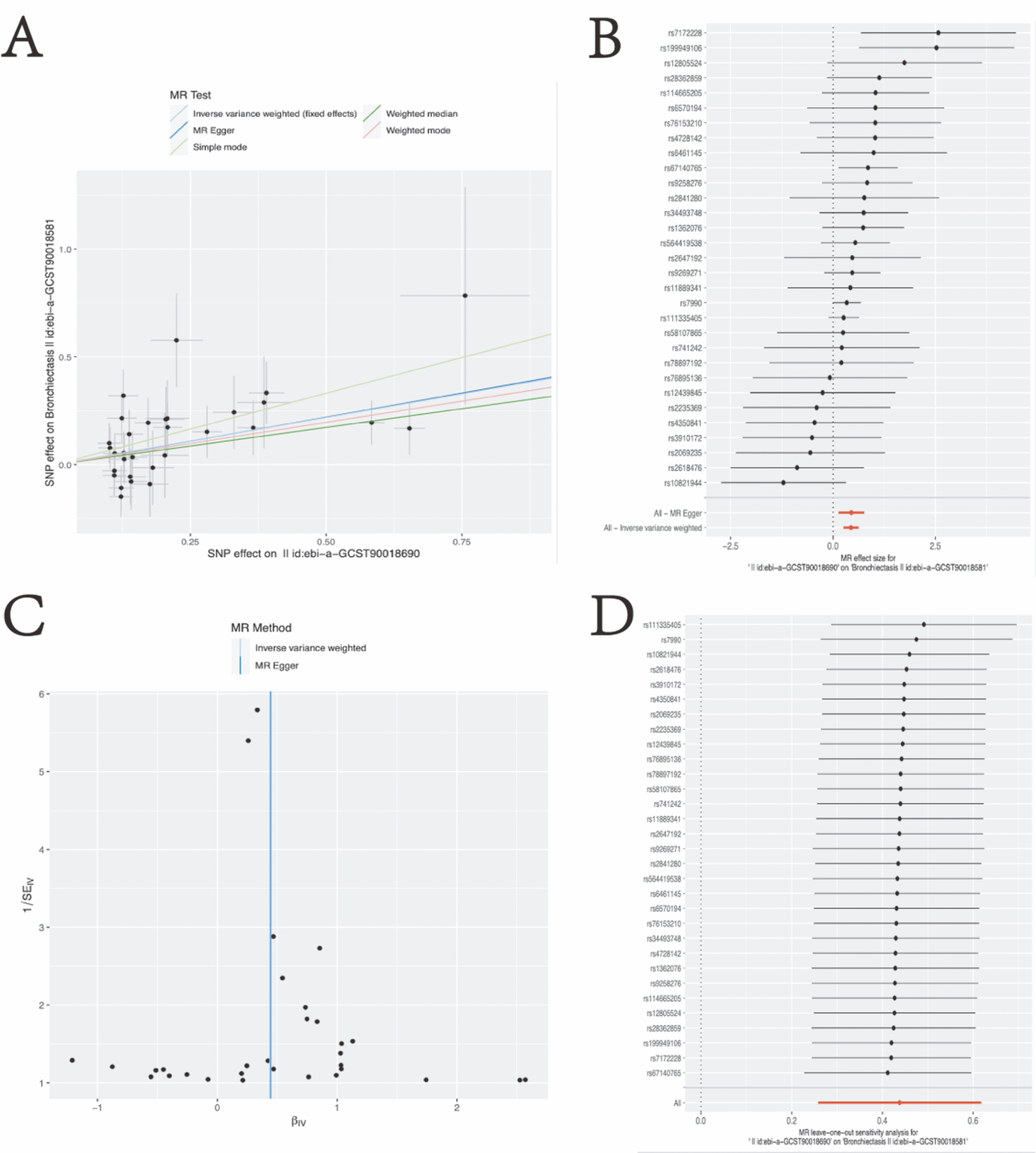


**Supplementary Figure 2. MR sensitivity analysis of RA on bronchiectasis in East Asian populations**

Scatter plot (A), forest plot (B), funnel plot (C) and leave-one-out sensitivity analysis (D) of the effect of RA on bronchiectasis

**
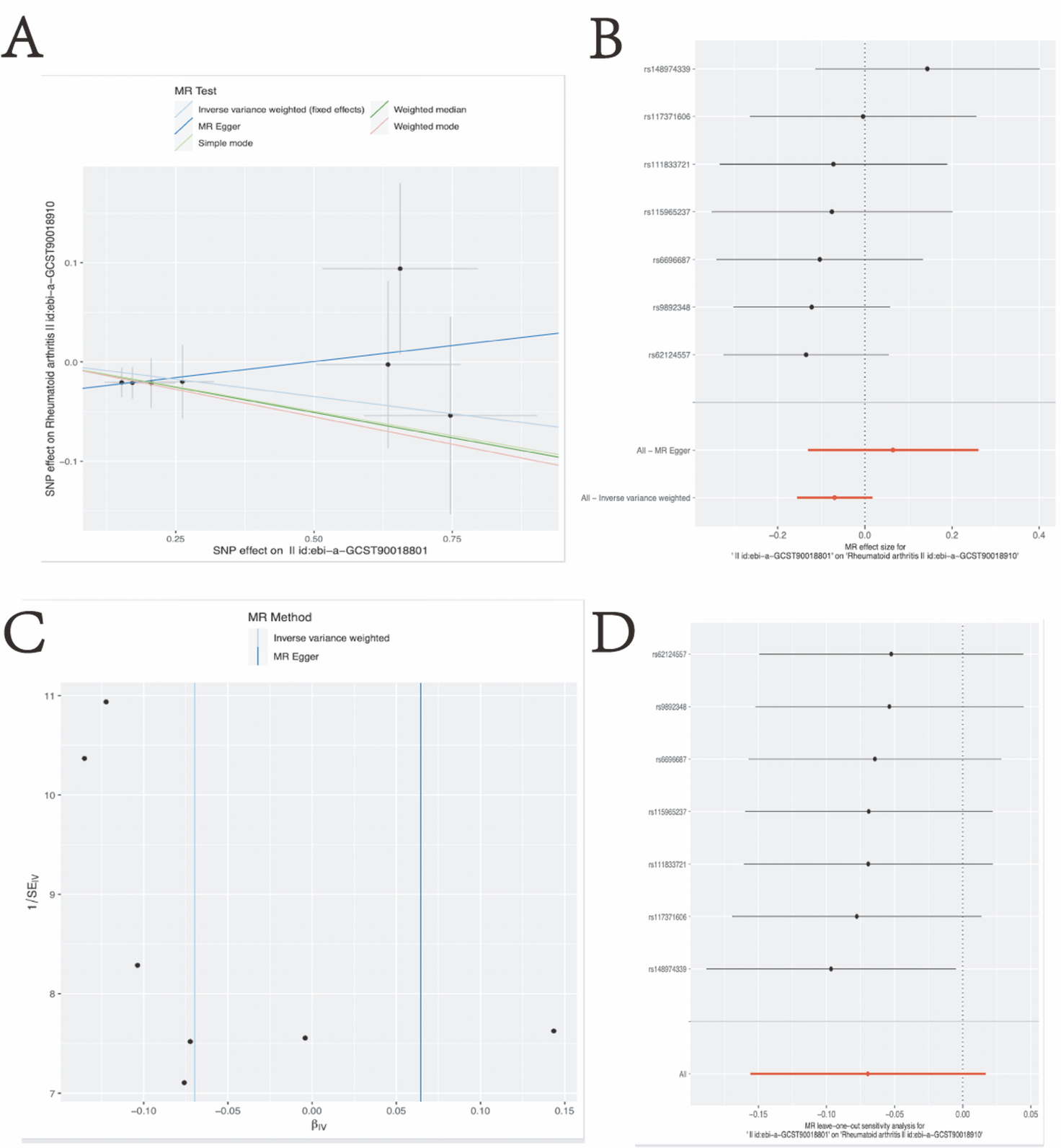
**

**Supplementary Figure 3. MR sensitivity analysis of bronchiectasis on RA in European populations**

Scatter plot (A), forest plot (B), funnel plot (C) and leave-one-out sensitivity analysis (D) of the effect of bronchiectasis on RA

**
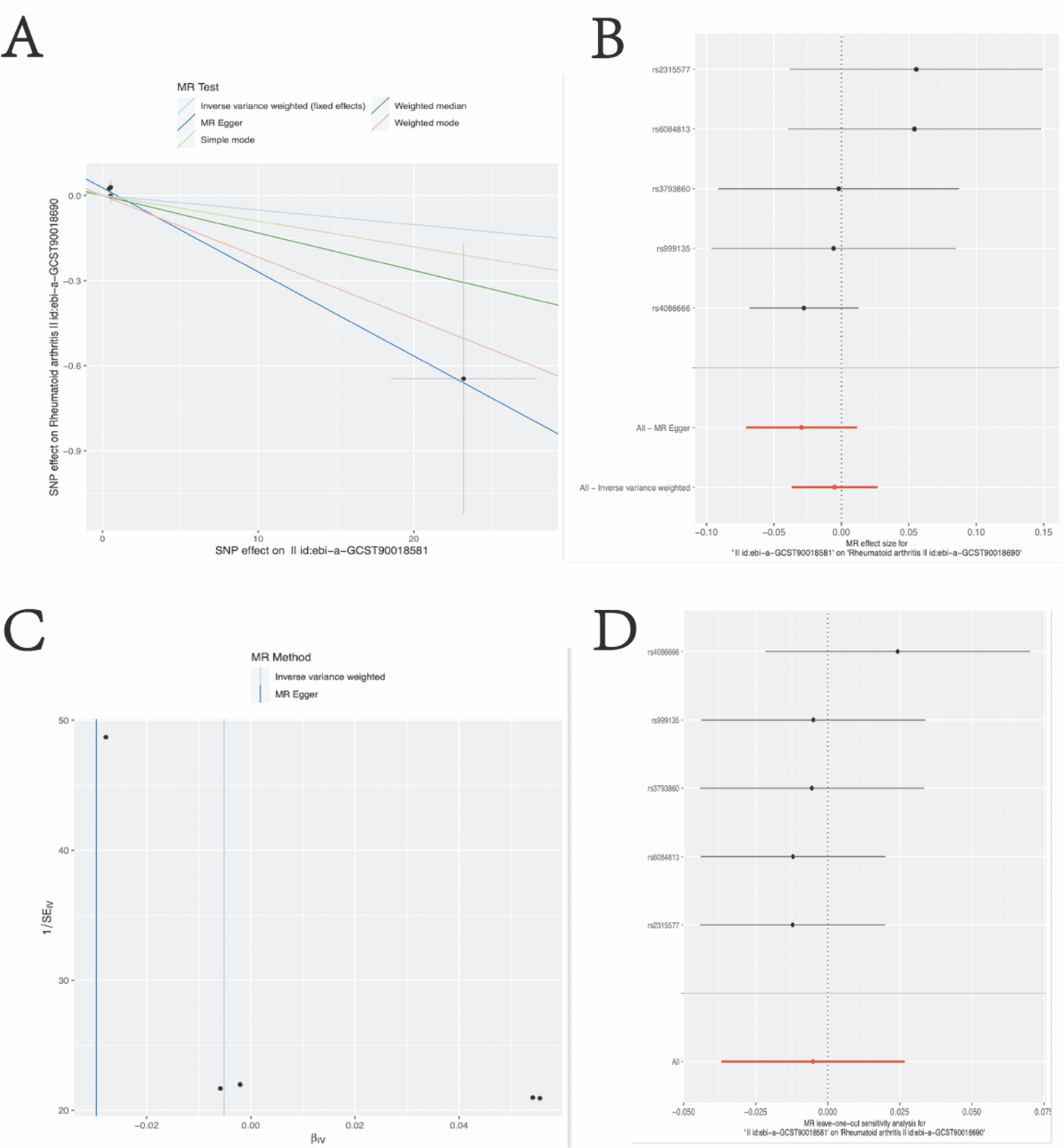
**

**Supplementary Figure 4. MR sensitivity analysis of bronchiectasis on RA in n East Asian populations**

Scatter plot(A), forest plot (B), funnel plot (C) and leave-one-out sensitivity analysis (D) of the effect of bronchiectasis on RA
